# The Diagnosis of Abdominal Emergencies in Children
*Based on a paper given to the Bristol Medico-Chirurgical Society on 9th April, 1952.


**Published:** 1952-07

**Authors:** R. E. Horton

**Affiliations:** Lecturer in Surgery, University of Bristol; Assistant Surgeon, United Bristol Hospitals


### THE DIAGNOSIS OF ABDOMINAL EMERGENCIES IN CHILDREN*

BY

R. E. HORTON, *M.B.E.*, F.R.C.S.

*Lecturer in Surgery, University of Bristol; Assistant Surgeon, United Bristol Hospitals*

Abdominal pain in children, although frequently of little significance, may sometimes be sinister and indicate the beginning of a serious pathological process. It is the fear of the consequences of an untreated acute abdominal emergency which makes the care of a child with abdominal pain one of anxiety and responsibility. The child who suffers from recurrent umbilical pain is in a position of particular danger, for the knowledge that previous pain has been of trivial significance and that it has resolved quickly and spontaneously may dull the sensibility of the observer; it is thus that acute appendicitis will progress under such a diagnostic mask as mesenteric adenitis until perforation occurs and general peritonitis is established.

Acute appendicitis is the most common surgical emergency of children, but in babies intussusception is an additional cause of severe abdominal colic. These two conditions, their variants, and the pitfalls which they may occasion, are the chief subject of discussion in this review.

It has rightly become the practice to regard the malignant tumours of infants and children as surgical emergencies. Some observations on the management of Wilms's tumour of the kidney are therefore not out of place in a paper devoted to the abdominal emergencies of children.

### ACUTE APPENDICITIS

Appendicitis may occur at any age, though it is rare under two years and uncommon under four years. It is more dangerous in children than in adults and a heavier penalty is paid for delayed or mistaken diagnosis. Localization of infection in the peritoneal cavity is far less effective in children, partly because the greater omentum is a relatively slender structure and is imperfect in surrounding and walling-off an area of inflammation. Thus children's appendicitis in its early stages differs little from appendicitis in adults, but there are no adhesions and the omentum is rarely found wrapped round the appendix. It is this failure of the omentum to set up a defence barrier which gives appendicitis in children its sinister reputation. The operation of appendicectomy is one of particular ease. But when perforation of the appendix takes place the whole peritoneal cavity is contaminated with pus.

Many children exhibit the classical sequence of symptoms: pain begins in the middle of the abdomen and later radiates to the right iliac fossa and vomiting

* Based on a paper given to the Bristol Medico-Chirurgical Society on 9th April, 1952.

or nausea is complained of after the pain has been present for some time. The pulse and temperature are elevated, the tongue is furred, and there is guarding and tenderness in the right iliac fossa. But unfortunately many cases do not show these characteristic features, or the sequence is atypical. In these the general practitioner is posed a most difficult problem of diagnosis. When the pathological process has been allowed to proceed unchecked for forty-eight or seventy-two hours the diagnosis is all too clear and the newly-qualified resident wonders how such delay could have been countenanced by an experienced practitioner.

But delay often occurs before the doctor is called in. We live in an age when a bottle of medicine is expected for every ill. The doctor who does not distribute medicine may lose his reputation among his patients. In the family circle many mothers still consider a right to control the bowels of their children is vested in them. Aperients are distributed and bowels are " turned out ". This does little harm to the children and much good to the manufacturers. Unfortunately the onset of abdominal pain may be greeted as an immediate indication to give a laxative. Castor oil used to be given, but syrup of figs is now more popular and many children with the abdominal pain of appendicitis have received at least one dose before the doctor is called in. In addition to the danger of perforation which this occasions, there is a period of delay while the effects of the dose are awaited.

Pain is the most common initial symptom of appendicitis. It may not be very severe, however, and in a few cases is not present at the onset. In these, vomiting, or less often, diarrhoea occurs before pain is complained of. Diarrhoea is well known in pelvic appendicitis, but is often present in children irrespective of the position of the appendix and may herald a particularly acute attack. In these cases there is considerable danger that a diagnosis of enteritis may be made in the early stages. Guarding is not a very reliable sign and is frequently absent, especially in cases in which the appendix is retrocaecal. But tenderness is always present and usually it is maximal somewhere in the right iliac fossa. The temperature is usually raised but in some cases it may be normal. Thus the combination of pain in the abdomen and tenderness in the right iliac fossa are the features which should excite a strong suspicion that a child has acute appendicitis, but it must be emphasized that in some cases the pain is not at all severe.

If the attack is allowed to proceed resolution may take place and will be accompanied by abatement of the symptoms. But it is more likely that the appendix will perforate. In some cases, particularly of pelvic appendicitis, perforation will be followed by the formation of a local abscess. There will be symptoms attributable to the position of the abscess. If it is in the pelvis there will be tenesmus and rarely retention of urine. At the same time the temperature becomes more elevated and the tongue more furred. Careful examination may disclose that a lump is present, and this is especially likely if the pelvic part of the peritoneal cavity is examined with a finger in the rectum, for the tone of the abdominal muscles may conceal a lump from abdominal palpation. In other cases perforation will be followed by general peritonitis. It is not always possible in looking back over the history of a patient admitted late and with general peritonitis, to say when perforation took place. There is no sudden episode of severe pain as in perforation of a peptic ulcer. On the contrary, the relief of tension inside the appendix which accompanies perforation may result in a considerable degree of relief from pain. Thus the symptoms of general peritonitis may be misjudged or not even recognized. The child becomes progressively more ill with fever, a dry furred tongue, and other signs of dehydration. The abdomen becomes gradually more distended and it is silent in consequence of paralytic ileus which accompanies peritonitis. Vomiting and constipation are present, though vomiting is not necessarily severe if the jejunum is not affected by the paralytic process. It is not common for children to be admitted in this condition, but a few are admitted to the Children's Hospital in the course of every year and some of these may die.

The diagnosis is easy in late cases, but those which confront the practitioner in the first twenty-four hours may tax his diagnostic skill to the utmost. Acute non-specific mesenteric adenitis is a common cause of abdominal pain and is the disease most often confused with appendicitis. There are two points of some value in the differential diagnosis. Mesenteric adenitis is usually accompanied by a nasopharyngeal infection; and the episodes of abdominal pain are frequently recurrent. In some cases there is no abdominal tenderness and then appendicitis can be excluded. But in other patients with the abdominal pain of mesenteric adenitis there is tenderness in the right iliac fossa and although the clinical picture is not identical with that of typical acute appendicitis the latter cannot be excluded. In these cases it is far safer to admit the child to hospital and submit him to an exploratory operation and appendicectomy than to await the development of the later stages of appendicitis.

### INTUSSUSCEPTION

This malady, far less common than appendicitis, is generally confined to infants of four to eighteen months. The typical clinical features of intussusception are well known. After a few hours or more of discomfort during which the baby whimpers and cannot sleep, severe abdominal colic begins. During the attacks of pain the baby usually draws up his legs and screams with pain. As the episode passes off he lies quite still and the face assumes a characteristic pallor. Blood is passed in the stool or stains the finger cot after rectal examination. Careful abdominal palpation discloses a mass in the epigastrium or less often on the left side of the abdomen. Many cases present such a history, but in others one or more of the classical signs may not be observed. The amount of bleeding depends on the tightness of the intussusception and it is absent in about fifty per cent of cases. The tumour may be concealed under the liver margin and then it will not be felt by the most careful observer. If there is a strong suspicion of intussusception the child should be admitted to hospital for observation. In many cases suspicion will be strong enough to justify laparotomy without completion of the classical triad of pain, tumour and blood in the stools. In some doubtful cases a barium enema may be used to establish the diagnosis but enemata should never be used therapeutically. In later cases there are signs of intestinal obstruction; constipation is absolute and vomiting severe. The abdomen is distended and shows the typical ladder pattern of distended small bowel. In these children the intussusception has been present for forty-eight hours or more. They are in very serious danger, probably with gangrene of the intussuscepted bowel.

Recurrence of intussusception occurs in about two per cent of cases. The mother may then recognize the symptoms and bring the baby to hospital with the diagnosis made, as happened recently at the Children's Hospital. In these recurrent cases a small bowel tumour may be present. When intussusception occurs in older children it is nearly always secondary to a benign or malignant neoplasm of the bowel. The diagnosis in these rare cases is difficult. There is colicky abdominal pain, evidence of small-intestine obstruction and possibly a palpable tumour.

### MECKEL'S DIVERTICULUM

No discussion on the surgical emergencies of children would be complete without a note on the complications of Meckel's diverticulum, for these are most likely to occur in early life. Blood in the stool is a common symptom in young children and infants, but when the bleeding is severe it probably comes from ulceration in a Meckel's diverticulum.

Meckel's diverticulum may invaginate into the ileum and become the apex of an intussusception. It may also be the site of acute inflammatory changes indistinguishable clinically from acute appendicitis. But the wide mouth of Meckel's diverticulum makes it less likely to become infected and indeed acute diverticulitis is very uncommon.

When the diverticulum remains attached to the umbilicus by a persistent cord, the child lives in danger of intestinal obstruction resulting from kinking of a loop of small bowel across the persistent band. In such cases there is nothing to guide the clinician as to the cause of an established intestinal obstruction. Moreover, if the obstruction is high there will be copious vomiting without abdominal distension, and then even the diagnosis of obstruction will be difficult.

### WILMS'S TUMOUR

It is now clear that the appalling results of treatment of this tumour can be improved if treatment by operation can be carried out immediately. The diagnosis is generally clear on clinical grounds, though an intravenous pyelogram may be indicated to confirm it. Nephrectomy should be performed on the day the diagnosis is established or as soon after as is possible. In this way, as little time as possible is left for pulmonary metastases to become established. There must be one day in which tumour cells are confined to the kidney and another in which the first vanguard of cancer reaches the lungs. The sooner operation is done the less likely is this to have taken place. Another danger is that of repeated examination. A Wilms's tumour is soft, cellular, and vascular. Repeated palpation of an interesting tumour by inexperienced students learning to feel an enlarged kidney invites the dissemination of malignant cells. The only certain way of stopping examination is to take the tumour out. So long as it is still in the patient it should be treated with the utmost delicacy and as urgently as a case of acute appendicitis, operation being delayed only long enough to ensure that blood is available for transfusion. Pre-operative radiation is not worth the delay which it causes and should only be given when the tumour is so large as to be thought inoperable. In these cases radiation causes shrinkage of the tumour and may make it removable, but cure is then very unlikely.

### CONCLUSION

The abdominal emergencies of children provide many problems of diagnosis. The most difficult problem concerns the significance of abdominal pain. It is more important to decide whether or not the pain is due to serious abdominal disease than to know its precise cause. Many children have typical symptoms but in others the signs are unusual and the history conflicting. While some of these may be watched safely at home, there is a risk in delaying their admission to hospital until advanced and dangerous pathological processes have occurred. If serious doubt exists advantage should be taken of the added facilities for observation which exist in hospital. Should serious abdominal disease then be confirmed surgical intervention can be arranged without further delay.

